# Multiple Sclerosis Patient-Specific Primary Neurons Differentiated from Urinary Renal Epithelial Cells via Induced Pluripotent Stem Cells

**DOI:** 10.1371/journal.pone.0155274

**Published:** 2016-05-09

**Authors:** Megan G. Massa, Barbara Gisevius, Sarah Hirschberg, Lisa Hinz, Matthias Schmidt, Ralf Gold, Nora Prochnow, Aiden Haghikia

**Affiliations:** 1 Neurologische Klinik der Ruhr-Universität Bochum, St. Josef-Hospital, Bochum, Germany; 2 Department of Neuroanatomy, Ruhr-Universität Bochum, Bochum, Germany; Julius-Maximilians-Universität Würzburg, GERMANY

## Abstract

As multiple sclerosis research progresses, it is pertinent to continue to develop suitable paradigms to allow for ever more sophisticated investigations. Animal models of multiple sclerosis, despite their continuing contributions to the field, may not be the most prudent for every experiment. Indeed, such may be either insufficient to reflect the functional impact of human genetic variations or unsuitable for drug screenings. Thus, we have established a cell- and patient-specific paradigm to provide an *in vitro* model within which to perform future genetic investigations. Renal proximal tubule epithelial cells were isolated from multiple sclerosis patients’ urine and transfected with pluripotency-inducing episomal factors. Subsequent induced pluripotent stem cells were formed into embryoid bodies selective for ectodermal lineage, resulting in neural tube-like rosettes and eventually neural progenitor cells. Differentiation of these precursors into primary neurons was achieved through a regimen of neurotrophic and other factors. These patient-specific primary neurons displayed typical morphology and functionality, also staining positive for mature neuronal markers. The development of such a non-invasive procedure devoid of permanent genetic manipulation during the course of differentiation, in the context of multiple sclerosis, provides an avenue for studies with a greater cell- and human-specific focus, specifically in the context of genetic contributions to neurodegeneration and drug discovery.

## Introduction

Though typically defined as an autoimmune demyelinating disease of the central nervous system, disease hallmarks of multiple sclerosis (MS) also include early-occurring, continuing axonal neurodegeneration and neuronal atrophy, both of which contribute significantly to later disease course [1]. While this neurodegeneration has been established as a byproduct of neuroinflammation, accumulating evidence indicates that the two processes can also be dissociated from one another, occurring in parallel via independent mechanisms [2]. However, despite this extensive characterization of disease course and symptomology, MS etiology remains unknown, though there is consensus that–as for other multifactorial diseases–genetic, epigenetic, and environmental factors together contribute to both disease onset and course [3]. In an attempt to further tease apart the contributions of each of these factors, it is important to note that gene expression and epigenetic profiles between the major cell types involved in MS, neurons and immune cells, may differ, and thus may contribute to disease etiology and course in unrelated, cell type-specific ways. Hence, an appropriate disease paradigm is required to investigate questions of specific contributions to MS.

Induced animal models, such as rodent experimental autoimmune encephalomyelitis, remain crucial to disease research; however, they contain many limitations in modeling human disease pathology. Because of this, they remain inappropriate for drug screenings, investigations into the plausible genetic contributions of a polygenic disease, and studies of specific neurodegenerative processes. A cell-specific study in MS is thus relevant to our basic understanding of the disease. In other disease contexts, such models have arisen in the form of patient-derived, human iPSCs, which can then be studied in their pluripotent state or in a subsequently differentiated form. iPSCs can be generated from a variety of somatic cells, including skin fibroblasts [4], keratinocytes [5], peripheral blood cells [6], and adipose stem cells [7]. Indistinguishable from embryonic stem cells in proliferation, morphology, and gene expression, iPSCs are typically induced using transcription factors Oct3/4, Nanog, c-Myc, and Klf4 [4]. Major advances have been made through the development of minimally-invasive collection techniques, namely the usage of easily-accessible cells such as peripheral blood mononuclear cells and renal cells [8]. In addition, integration-free transfection techniques, such as electroporation with episomal plasmids, have been developed to avoid viral-mediated insertional mutations. While these methodologies typically result in a lower transfection efficiency [9], vector integrations come at the expense of potential interference with the functionality of iPSC derivatives. These derivatives, specifically neuronal forms, have been particularly useful for neurodegenerative diseases, including Parkinson’s disease (PD), Alzheimer’s disease (AD), and amyotrophic lateral sclerosis (ALS), where current animal models have led to limited translational success [10–21]. Furthermore, it is important to note that these neurodegenerative diseases, as is the case with MS, may be contributed to by external environmental factors inducing alterations in cellular epigenetic profile. Because there is evidence of both genomic stability [15,22,23] and epigenetic persistence [24] through the procedure of iPSC conversion and differentiation, the cell- and patient-specific *in vitro* paradigm may not only contain relevant genetic material, but also epigenetic information necessary for the investigation of disease etiology and biochemistry.

Herein, we describe the successful conversion of non-invasively obtained human renal proximal tubule epithelial cells to MS patient-specific primary neurons via an iPSC procedure. The establishment of such a procedure can allow for greater understanding of human cellular mechanisms of MS, potentially leading to novel therapeutic targets and subsequent efficacious drug discovery.

## Materials and Methods

### Acquisition of urine samples

It is estimated that 2,000–7,000 renal tubule cells pass through urinary excrement daily [25], allowing for a non-invasive method for collection of fibroblast-like cells. After written consent, a male MS patient affiliated with the St. Josef-Hospital–Klinikum der Ruhr-Universität (32 years old, RRMS, EDSS 2, taking no MS-related medication) and female healthy control (25 years old) provided urine samples in accordance with the guidelines dictated by the Ethics Committee of the Ruhr-Universität Bochum (register number: 4745–13); said ethics committee specifically approved this study. Each participant was provided with Octenisept^®^ sterilization liquid (Schülke & Meyer), a sterile beaker, and instructions on sterile collection method.

### Plasmid isolation

Three episomal vectors were utilized for reprogramming, as per previous reports of fibroblast transfection success [26]: pCXLE-hOCT3/4-shp53, pCXLE-hSK, and pCXLE-hUL (Addgene Plasmid #27077, #27078, and #27080, respectively). Plasmid-containing *E*. *coli* were cultured, and colonies picked and expanded in LB Medium with ampicillin. Plasmid extraction was accomplished through the PureYield^TM^ Midiprep System (Promega) as per the manufacturer’s instructions.

### Isolation and culture of Renal Proximal Tubule Epithelial Cells (RPTECs)

**Fig 1:**
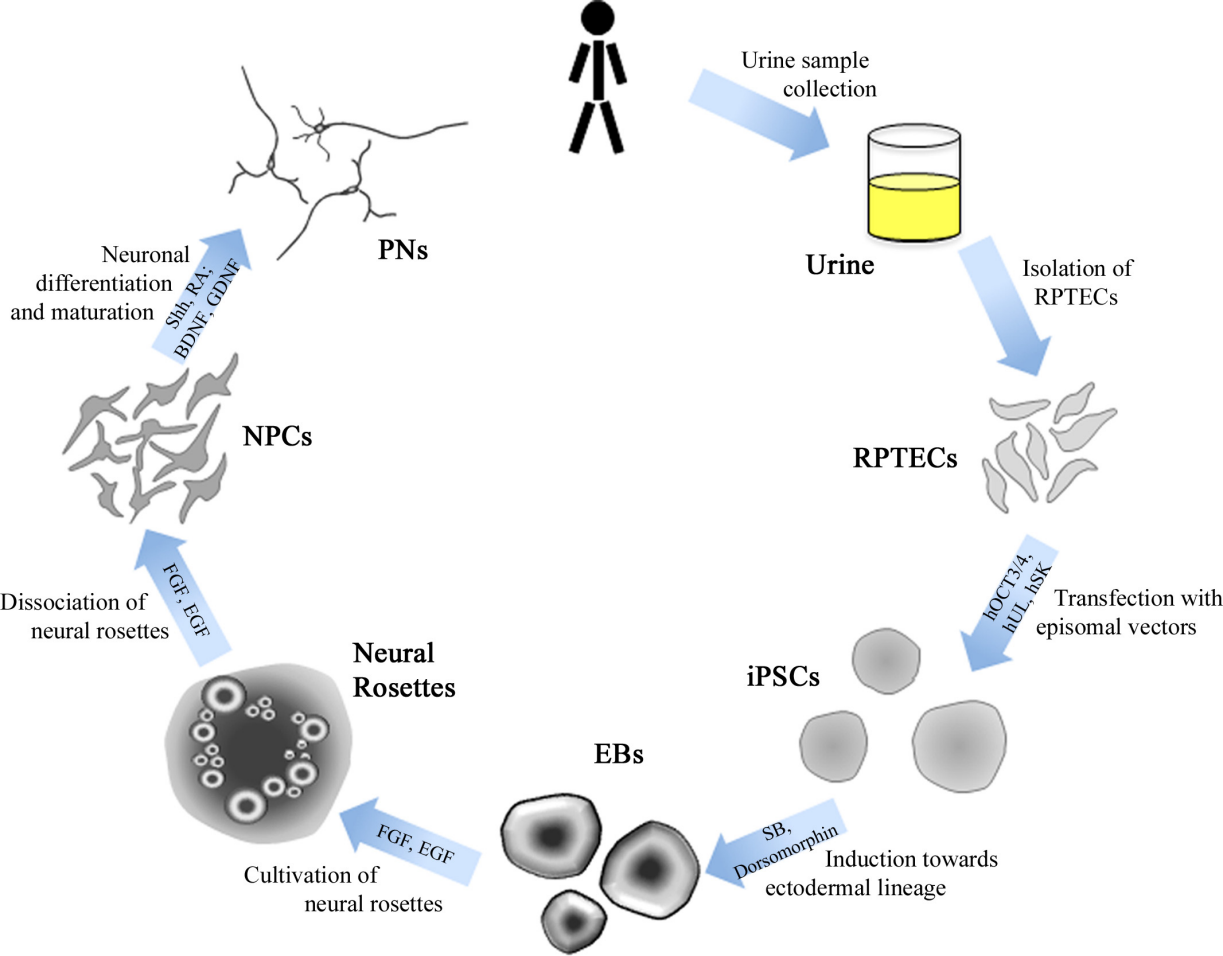
Schematic overview of methodological procedure. After isolation and cultivation of RPTECs from urine samples, cells were transfected with pluripotency-inducing genes hOCT3/4, hSK, and hUL, resulting in long-lasting iPSC colonies. Through the cultivation of iPSCs in a free-floating condition, embryoid bodies (EBs) were formed and guided towards ectodermal lineage by addition of SB431542 (SB) and dorsomorphin for the suppression of the meso-, ento-, and epidermal lineages. Maturations of these cell aggregations with EGF and FGF under adherent conditions resulted in neural rosette formations, which were subsequently excised and dissociated into neural progenitor cells (NPCs). These cells were then differentiated to primary neurons (PNs) via basal medium with Sonic hedgehog (Shh) and retinoic acid (RA); cultures were further cultivated through the addition of BDNF and GDNF.

A schematic overview of these entire methodology is depicted in **Fig 1**. The procedure for isolation of RPTECs was derived from Zhou et al. [8] with various modifications.

Following aspiration of urine supernatant after the first isolation centrifugation, 10 mL washing buffer was utilized to resuspend and consolidate all pellets. Due to contamination inherently present in urine samples, cells were washed a second time before a final isolation centrifugation. Pellets were then resuspended in warmed RE proliferation medium (REGM^TM^; Lonza) prior to drop-wise transfer into two wells of a cell culture-treated, 0.1% gelatin-coated 12-well plate. To further reduce collection contamination, 100 μg/mL Primocin^TM^ (InvivoGen) was added for the first 5 days (D0-D4) of culture. On D1, the addition of 1 mL REGM to each well occurred without previous aspiration of old medium as per the original protocol. From D2 onward, REGM was completely changed every day, and cells were washed with Dulbecco’s Phosphate Buffered Saline with calcium and magnesium (DPBS+/+; Life Technologies) every even day before replacement of REGM. When wells reached 90% confluence (approximately two to three weeks after plating), cells were passaged through an initial wash with DPBS without calcium and magnesium (DPBS-/-; Life Technologies) and subsequently trypsinized with TrypLE^TM^ Express (Life Technologies), the reaction being terminated via dilution. Cells were centrifuged and resuspended in REGM for plating onto a cell culture-treated, 3.5 cm, 0.1% gelatin-coated plate. Plates received a full medium-change every day and were washed with DPBS+/+ every other day beginning with D2 post-trypsinization. Cells were passaged a second time to a 10 cm plate in order to cultivate the appropriate number of cells required for transfection (typically 80–90% confluence). All centrifugations were carried out at 450 x *g* for 10 minutes at room temperature (RT).

### Transfection of RPTECs into iPSCs

RPTEC cultures were incubated for one hour with 10 μM Y-27632 ROCK-inhibitor (Selleckchem) prior to DPBS-/- wash and subsequent trypsinization. Plasmid concentrations of 0.88 μg per 1 million cells were utilized. Transfection was achieved through electroporation via the Neon^®^ Transfection System (Life Technologies) as per the manufacturer’s instructions, and settings were programmed to one, 30 ms pulse at 1,100 V. Transfected cells were seeded on cell-culture-treated, Matrigel^®^-coated (Corning Life Sciences) plates filled with Fibroblast medium [27] and incubated with 10 μM ROCK-inhibitor for the first twenty-four hours. Medium was changed every odd day beginning with D1 post-transfection. When cells reached 70% confluence, the medium was switched to TeSR^TM^-E7^TM^ reprogramming medium (Stemcell Technologies) until first colonies appeared; cutting and passage of colonies occurred in accordance with manufacturer’s instructions using mTeSR^TM^1 medium (Stemcell Technologies), typically every 5–7 days.

### Formation of embryoid bodies (EBs), rosette structures, and neural progenitor cells (NPCs)

iPSCs selected for EB formation were pre-incubated with 10 μM ROCK-inhibitor and subsequently washed with 20% KSR medium in DMEM/F12+Glutamax [28]. Three-dimensional structures were cultivated by scratching iPSCs from the plate using a cell scraper. Resulting EBs were maintained for 6 days on non-culture-treated, un-coated dishes in mTeSR^TM^1 medium, shaking twice daily. Full medium changes occurred every two days, with medium being supplemented with 10 μM SB431542 (Biozol) and 5 μM dorsomorphin (Sigma) on days 2 and 4. On day 6, EBs were plated onto cell culture-treated plates double-coated with 0.002% Poly-L-ornithine (PORN; Sigma) and 10 μg/mL laminin (Sigma) filled with either ITSFn or NSCM medium [29]. Neural rosette structures were mechanically isolated and NPCs cultivated as previously described [29], omitting prior neurosphere propagation.

### Induction of NPCs into primary neurons (PNs)

Confluent NPCs were re-plated onto cell culture-treated, PORN/laminin-coated 3.5 cm dishes, with 100,000 cells per dish (trypsinization for transfer halted with 20% KSR). Cells were incubated with NSCM [29] for 24 hours before being switched to differentiation medium (DMEM/F12+Glutamax, 2x N2 supplement, 2x B27 supplement, 50 μg/mL apo-transferrin, 200 μM ascorbic acid), with half medium changes every other day. Sonic hedgehog (500 ng/mL) and retinoic acid (4 μM) were supplemented to the medium for 6 days [29]; 10 ng/mL BDNF and 20 ng/mL GDNF from day 7 onward.

### Immunofluorescent staining

Cells of interest were plated onto appropriately-coated cover slips that had been previously cleaned with HCl and stored in EtOH. iPSC stainings were accomplished via a 10-minute 4% paraformaldehyde fixation and 0.5 nM ammonium chloride + 0.25% Triton X-100 (Sigma) antigen unmasking followed by a one-hour blocking period with 5% BSA in PBS; NPC and PN stainings via a 5-minute fixation with ice cold methanol followed by a 15-minute antigen awakening (3-minute for PNs) with 0.3% Triton X-100 in PBS and subsequent block using 5% BSA in PBS. In all cases, primary antibodies were incubated with cells overnight at 4°C, secondary antibodies in the dark for one hour at room temperature (see **Tables 1** and **2** for primary and secondary antibodies, respectively, and their dilution factors). For TH staining, an intermediate, biotinylated antibody was applied for one hour at room temperature prior to Alexa Fluor antibody incubation. All antibody dilutions were performed using 0.8% BSA in PBS.

**Table 1:** Primary antibody list and specifications.

	Antibody	Dilution	Host	Provider
**iPSCs**	Nanog (M-155)	1:100	Rabbit	Santa Cruz
	Oct4 (C-10)	1:100	Mouse	Santa Cruz
	Sox2	1:100	Rabbit	Bioscience
	SSEA4 (813–70)	1:100	Mouse	Santa Cruz
	Tra-1-60	1:100	Mouse	Santa Cruz
	Tra-1-81	1:100	Mouse	Santa Cruz
**NPCs**	Pax6	1:50	Goat	Santa Cruz
	Doublecortin	1:100	Goat	Santa Cruz
**PNs**	β-III-Tubulin (1)	1:10,000	Rabbit	Sigma
	β-III-Tubulin (2)	1:400,000	Mouse	Biolegend
	Neurogenin	1:50	Rabbit	Santa Cruz
	SMI-32R	1:10,000	Mouse	Covance
	ChAT	1:500	Rabbit	Abbexa
	TH	1:500	Sheep	Millipore
	GFAP cocktail	1:400,000	Mouse	BD Bioscience
	MBP	1:1000	Mouse	Biolegend
	O4	1:70	Mouse	Graciously provided by Dr. Andreas Faissner

**Table 2:** Secondary antibody list and specifications.

	Antibody	Dilution	Host/Antigen	Provider
**iPSCs**	Alexa 555	1:1000	Goat/anti-mouse	Life Technologies
	Alexa 555	1:1000	Goat/anti-rabbit	Life Technologies
**NPCs**	Alexa 488	1:1000	Rabbit/anti-goat	Life Technologies
**PNs**	Alexa 488	1:1000	Goat/anti-rabbit	Life Technologies
	Alexa 555	1:1000	Goat/anti-mouse	Life Technologies
	Alexa 488	1:1000	Goat/anti-mouse	Dako
	Biotinylated	1:1000	Rabbit/anti-sheep	Vector Laboratories

### Microscopy

Bright field images were captured with the cell^F program and accompanying Olympus IX51 microscope/camera system; immunoflorescent images with cellSens program and Olympus XM10. Quantification of stained PNs was accomplished by manual cell counting within 4–5 randomly-selected visual fields at 10x magnification from 3–4 independent experiments. Average field contained 93 DAPI-positive nuclei.

### Electrophysiological analysis of PNs

All cells underwent electrophysiological characterization at room temperature (~20°C) between 20 to 30 days post-differentiation. Cells cultured on PORN/laminin-coated cover slips were transferred into a recording chamber mounted on an inverted microscope (Zeiss Axiovert) and continuously superfused at a rate of 4 mL/min with oxygenated external solution (ACSF) containing (in mM): 124 NaCl, 2.7 KCl, 1.25 KH_2_PO_4_, 26 NaHCO_3_, 2 MgSO_4_, 2 CaCl_2_, and 10 glucose [30,31]. Recording pipettes were pulled from borosilicate glass and filled with internal solution containing (in mM): 130 potassium gluconate, 2 sodium gluconate, 20 HEPES, 4 MgCl_2_, 4 Na_2_ATP, 0.4 NaGTP, and 5 EGTA to reach a final impedance of 5–8 MΩ. Isolation of sodium currents was accomplished using a cesium-based internal solution containing (in mM): 130 cesium methanesulfonate, 2 NaCl, 20 HEPES, 4 Na_2_ATP, 0.4 NaGTP, and 5 EGTA. Whole-cell patch clamp recordings both in voltage and current clamp modes were carried out using a PC 501-A amplifier (Warner Instruments). Signals were filtered by a Humbug noise eliminator (Digitimer Ltd.) and digitized at a sampling rate of 10 kHz with WinWCP software (Strathclyde Inst. of Pharmacy and Biomedical Sci.). Recorded potentials were corrected for the liquid junction potential (10.3 mV).

To determine steady-state current-voltage relationships, recorded cells were voltage clamped to -60 mV and stimulated with 50 ms voltage steps (from -80 to +30 mV in 10 mV increments). Current responses were measured as the mean current amplitude during the last 25 ms of the voltage steps. For pharmacological characterization of fast inward currents induced by depolarizing voltage steps carried by voltage-dependent sodium channels, a hyperpolarizing voltage step (to -80 mV, 50 ms) followed by depolarizing voltage step (from -20 to 0 mV, 50 ms) was applied every 15 s while 10 μM tetrodotoxin (TTX) was bath-applied through the superfusion system. The firing behavior of cells was characterized in current clamp mode by increasing the holding current to change from subthreshold (approx. -70 mV) to suprathreshold (approx. -20 mV) membrane potentials.

## Results

### MS patient RPTECs can be isolated and transfected into iPSCs

**Fig 2:**
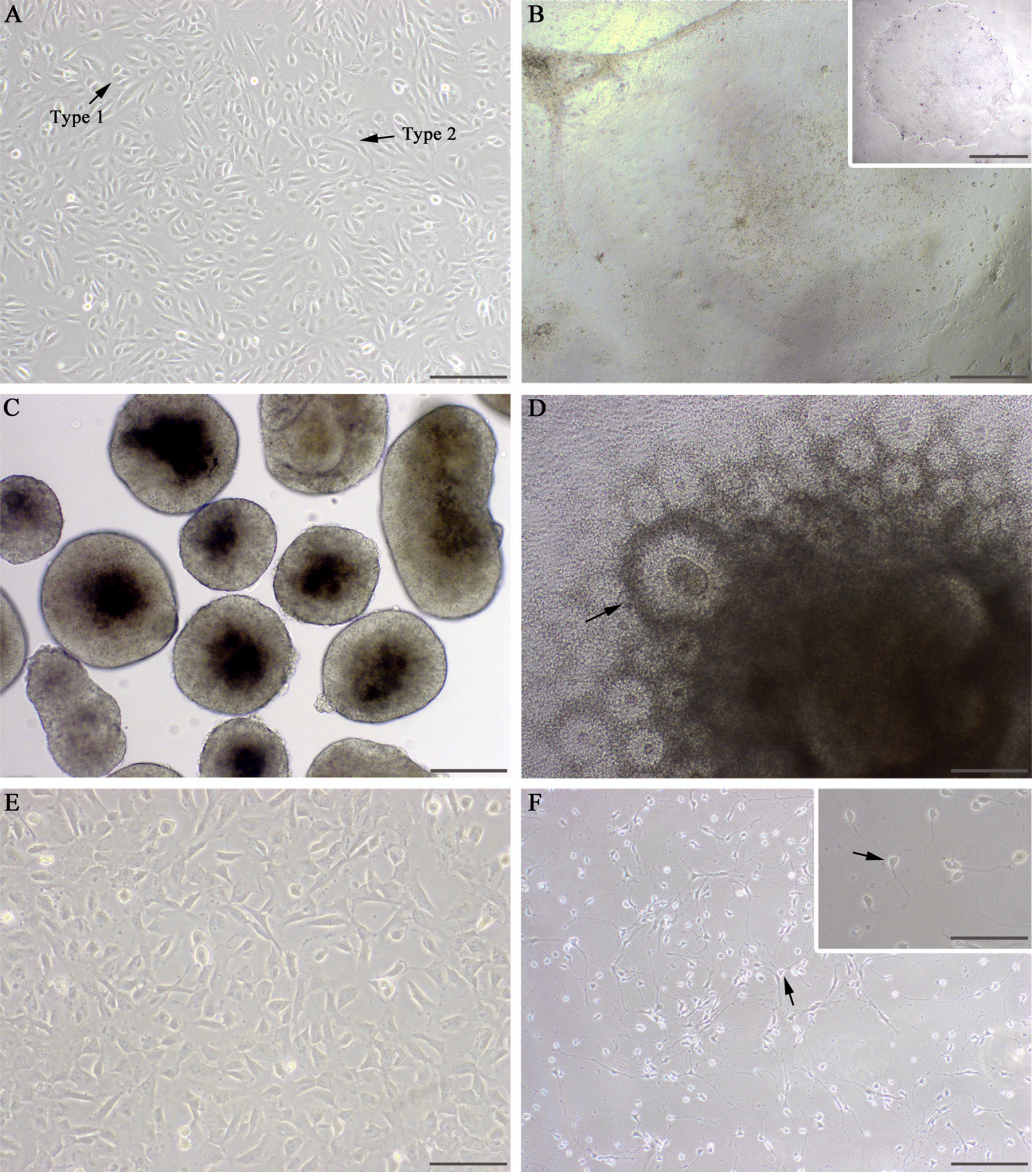
Examples of key stages in neuronal differentiation from epithelial cells. (A) Male patient RPTECs were photographed after 22 days *in vitro* and subsequently transfected; scale bar 500 μm. Arrows indicate distinct morphologies as previously reported [8]. (B) First fully-formed colonies were visualized and cut 21 days after transfection; scale bar 500 μm. Inset shows isolated colony at passage 19; scale bar 375 μm. (C) Cultured EBs were obtained from iPSCs after 4 passages; scale bar 200 μm. (D) Large neural rosettes were photographed and subsequently mechanically isolated 12 days after EB plating; scale bar 200 μm. (E) Cut rosettes underwent trypsinization to form a single-cell suspension of NPCs. Image shows NPCs 8 days after single-cell plating; scale bar 100 μm. (F) Induced primary neurons demonstrates typical neuron morphology, including pyramidal somata (indicated by arrows), extended axons, and formation of neural networks 21 days after switch to differentiation medium; scale bar 200 μm. Inset shows enlarged example of pyramidal morphology; scale bar 100 μm.

Cells procured from the urine samples of an MS patient and healthy control were successfully cultured, spending an average of 24 days *in vitro* prior to transfection. Most cell loss occurred prior to first passage and was due to contamination from the original urine sample, which was present in all cases. Upon advanced proliferation, dishes contained cells displaying two distinct morphologies (**Fig 2A**, arrows) as described previously [8]. RPTEC culture and transfection efficiency were not affected following rapid thaw from a cryopreserved state.

**Fig 3:**
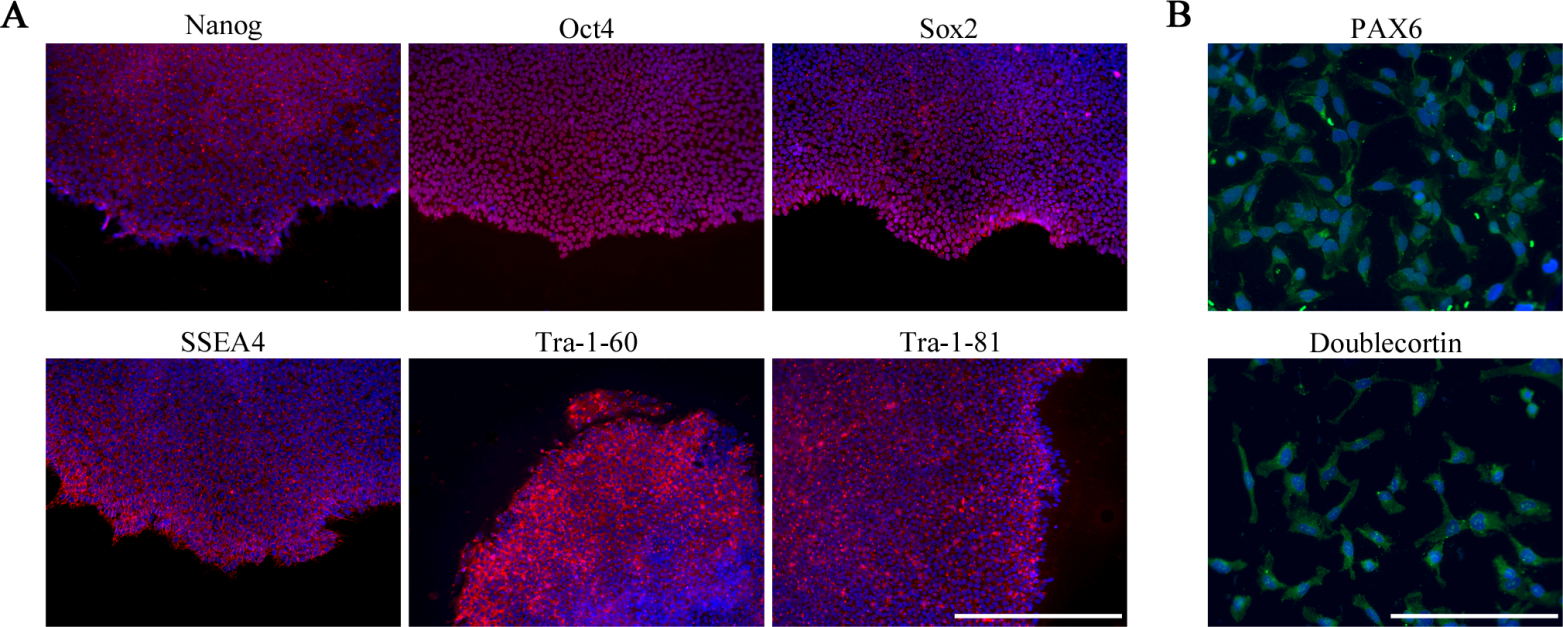
Staining of relevant markers confirm cellular identity of induced cells at iPSC and NSC stages. (A) Representative staining of MS patient-derived iPSC colony confirms the presence of various pluripotency markers: stem cell transcription factors Nanog, Oct4, & Sox2 and human embryonic stem cell surface markers SSEA4, Tra-1-60, and Tra-1-81. Cells acquired were stained at passage 6; scale bar 100 μm. (B) Male MS patient NPCs were obtained via single-cell suspension following mechanical isolation of neural rosette structures. Cells stained positive for neurogenesis transcription factor PAX6 and neuronal precursor microtubule-associated protein doublecortin 8 days and one passage after rosette dissociation; scale bar 50 μm.

Three successful, independent transfections were performed using MS patient RPTECs, two using healthy control cells, with viable colonies forming in 83.3% of all transfection attempts. The first viable colonies appeared at an average of 25 days post-transfection, in line with the mTeSR^TM^-E7^TM^ and mTeSR^TM^1 manufacturer protocol (**Fig 2B**). iPSCs (passage 4+) stained positive for all tested pluripotency markers: stem cell transcription factors Nanog, Oct4, and Sox2, along with human embryonic stem cell surface markers SSEA4, Tra-1-60, and Tra-1-81 (patient cells highlighted in **Fig 3A**).

### Specific pathway blockage and promotion leads to differentiation of patient-specific, RPTEC-derived iPSCs into NPCs

For the appropriate primary neuron differentiation, EB formations selective for ectodermal lineage were formed via the inhibition of endomesodermal processes (**Fig 2C**). This was accomplished using SB431542 and dorsomorphin to inhibit the activin/nodal and bone morphogenetic protein pathways, respectively [32]. Resulting EBs formed neural tubule-like rosette formations when plated and cultured with appropriate medium (**Fig 2D**). NPCs resulting from single-cell suspensions of rosette structures (**Fig 2E**) were confirmed as such via positive stainings for neurogenesis transcription factor PAX6 and neuronal precursor microtubule-associated protein doublecortin (**Fig 3B**).

### Induced PNs demonstrate typical morphology and TTX-dependent electrophysiological properties

**Fig 4:**
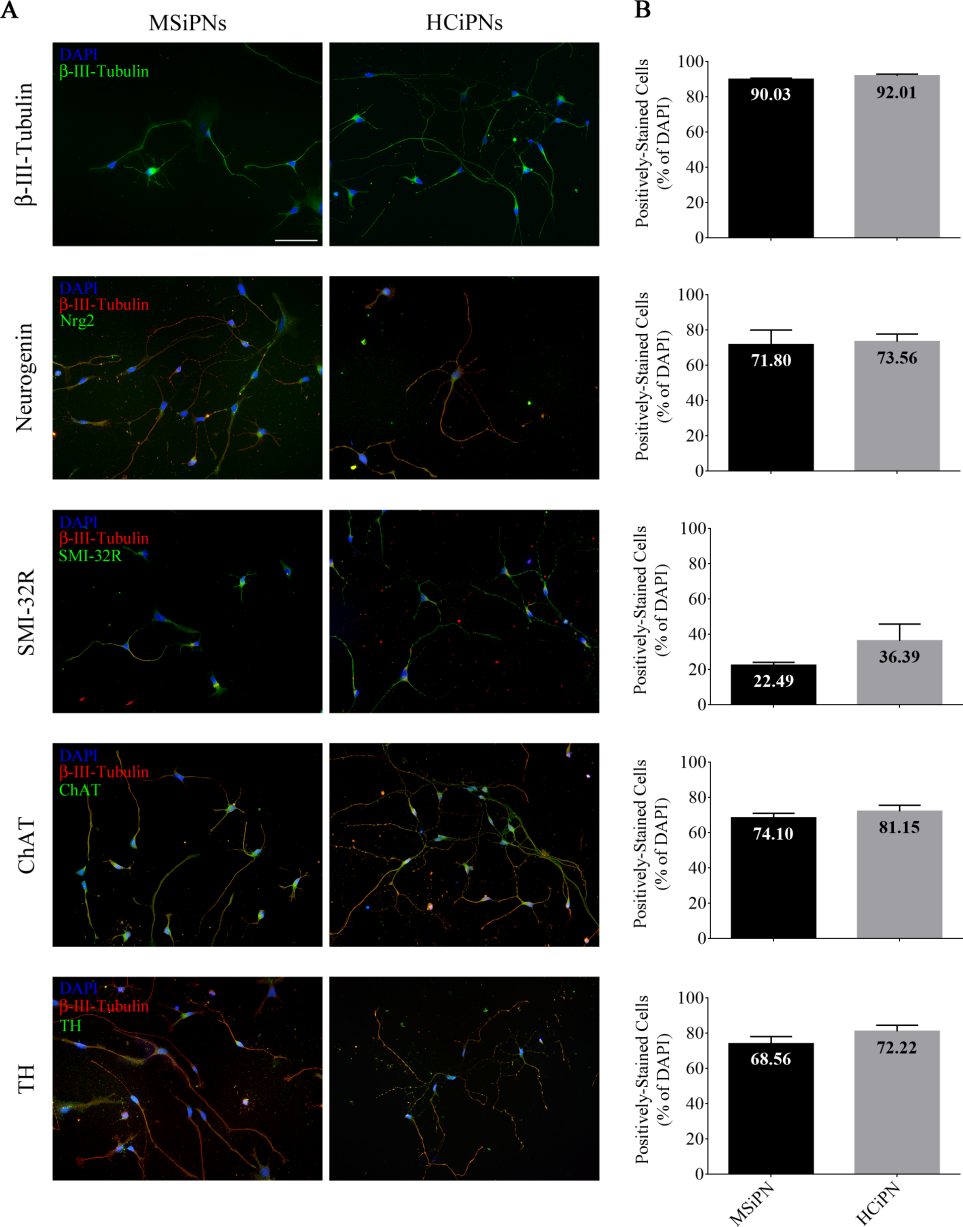
Characterization of HC and MSiPNs. (A) Cells were stained after an average of 20.5 days in differentiation medium (final cell count of 15,000 cells per well; staining begun 2 days following second plating); scale bar 50 μm. Cells stained positive for neuronal-specific cytoskeletal markers β-III-tubulin and SMI-32R, as well as neural transcription factor neurogenin (Nrg2). Cells also displayed less-intensive expression of neurotransmitter enzymes ChAT and TH. (B) Quantification of four to five randomly-selected visual fields of three or four independent experiments confirmed purity of neuron culture: 90.03% (92.01%) of patient (healthy control) cells in selected visual fields stained positive for β-III-Tubulin, 71.80% (73.56%) for tubulin + neurogenin, and 22.49% (36.39%) for tubulin with mature neuron marker SMI-32R, indicating a mix of neuronal developmental stages. ChAT and TH enzymes were also present in high levels, at 74.10% (81.15%) and 68.56% (72.22%), respectively. Error bars depict SEM.

Eleven individual PN differentiations from NPCs were performed for each participant. Modified differentiation protocol [29] resulted in a hyper-populated reservoir of immature neuron structures. Upon separation from the reservoir (80,000–100,000 cells per 3.5 cm dish), MS-patient induced (MSi)PNs displayed typical morphology (**Fig 2F**) and showed protein expression of both neuronal differentiation transcription factors and β-III-Tubulin & SMI-32R neuronal cytoskeletal markers (**Fig 4A**, overview images provided in **S1 Fig**) in the relative absence of GFAP (percent mean ± SEM: MSiPN 3.63 ±1.22, HCiPN 0.13 ± 0.13), myelin basic protein (MBP), and O4 (**S2 Fig**). Quantification of four to five randomly-selected visual fields within three or four separate stainings revealed a high neuronal purity in both patient and healthy control cultures, with 90.03 ± 0.66 and 92.01 ± 0.99 percent (± SEM) of cells, respectively, staining for β-III-Tubulin, 71.80 ± 8.33 & 73.56 ± 4.25 for tubulin and neurogenin, and 22.49 ± 1.68 & 36.39 ± 9.50 for tubulin and SMI-32R (**Fig 4B**). Characterization of cells revealed expression of both ChAT (68.56 ± 2.57, 72.22 ± 3.49) and TH (74.10 ± 4.07, 81.15 ± 3.43) in control and patient cells.

**Fig 5:**
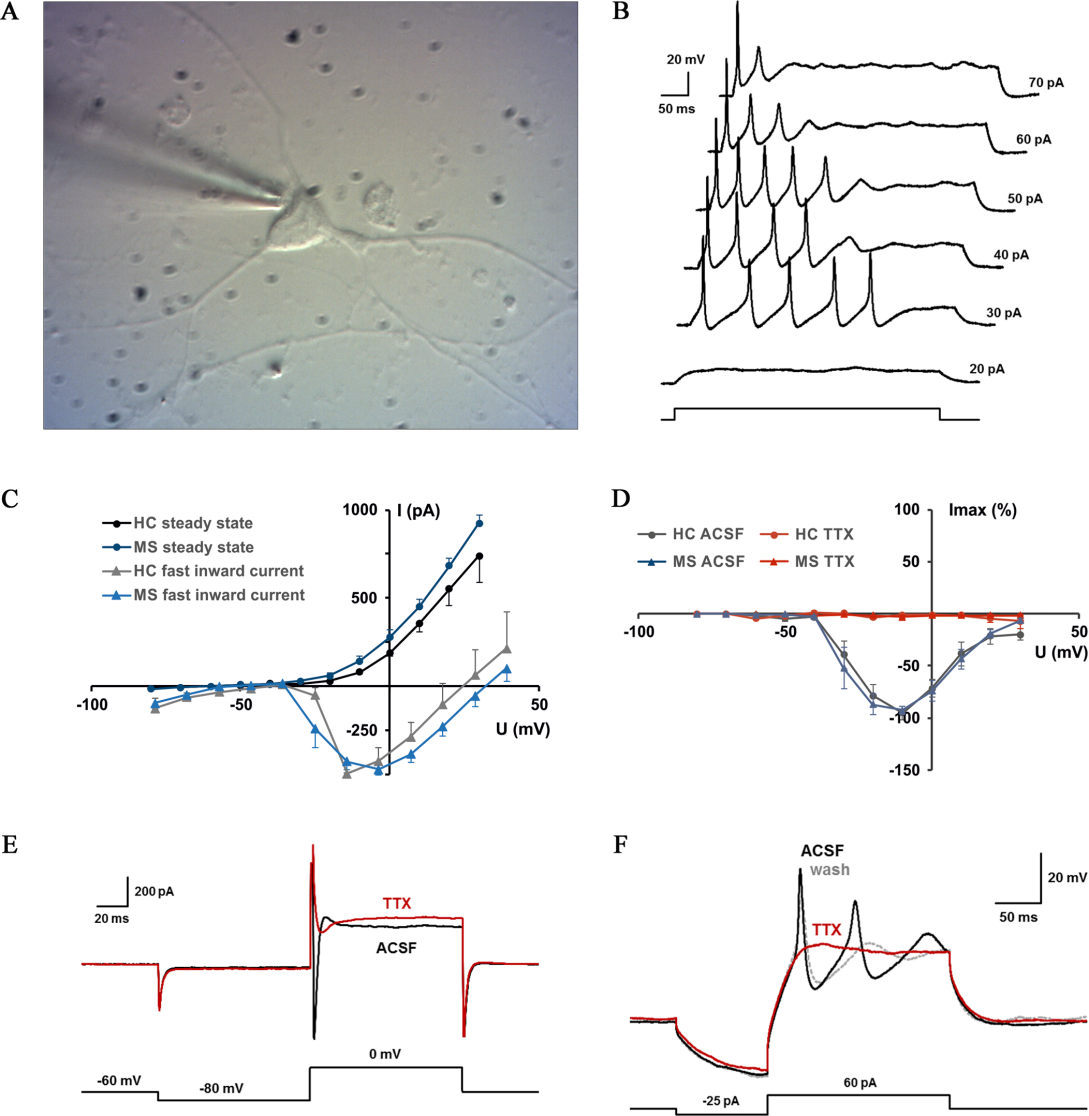
Electrophysiology of MSiPNs. (A) Representative photomicrograph of an MSiPN while advancing the recording pipette. Independent from the mode of culturing, examined cells were characterized by a pyramidal shaped soma and multipolar dendrite formations. (B) AP firing induced in MSiPNs by increasing depolarizing current injections. (C) Voltage dependence of steady state and fast inward currents recorded in cells derived from a healthy control (HC; 5 cells analyzed) and a MS patient (MS; 5 cells analyzed). (D) Isolated fast inward sodium currents in both MS- and HCiPNs are abolished by TTX bath (10 μM). (E) Representative MSiPN responses to TTX-block of sodium currents to depolarizing voltage steps and of (F) action potentials elicited by current injections.

In order to investigate the functional integrity of MSiPNs, whole-cell patch clamp recordings were performed in the voltage and current clamp mode *in vitro*. All investigated cells were characterized by pyramidal somata and multipolar dendritic morphology, as represented in **Fig 5A**. Depolarizing current injections induced single or multiple action potentials (APs) leading to irregular, fast-adapting firing in MSiPNs, with a mean duration of initial action potentials of 5.5 ± 0.2 ms (mean ± s.d.; **Fig 5B**). As expected from functional neurons, increases of current amplitude increased the firing rate (as indicated by reduced interspike intervals) until reaching depolarization block, thus suppressing AP firing (**Fig 5B**).

I-V curves achieved via voltage steps from -80 mV to +30 mV showed MSiPN steady state- and sodium current-relationships mimicked responses of those derived from healthy controls (HC; **Fig 5C**). Application of TTX to MSiPNs appropriately altered isolated fast sodium current-voltage relations (**Fig 5D**), also blocking both inward sodium currents under voltage clamp conditions and APs under current clamp conditions (**Fig 5E** and **5F**, respectively).

## Discussion

We have successfully derived functional, MS patient-specific primary neurons via non-invasive collection of renal proximal tubule epithelial cells. The development of such constitutes, to our knowledge, the first reliable *in vitro* human model of MS neurons, decisively augmenting the growing body of humanized, neurodegenerative disease models seen in recent years [18,19].

The limiting factor within the current procedure remains the RPTEC culture success rate. Contamination complications typically occurred within the initial culture, with rates being higher than those previously described [8]. However, because cellular collection is non-invasive, repeated collections are plausible, have no foreseeable deficit to patients. Despite this, the following procedure can be accomplished with alarming efficiency, with transfections resulting in hESC-like colonies and embryoid body attachments consistently developing neural tubule-like formation [29]. The manual isolation and single cell suspension of these cultures resulted in neural precursor cells that, when subjected to neuron-selective conditions, led to the creation of over-populated, neural “mother cultures”. Such has never before been reported, with these cultures acting as unusually long-lasting reservoirs for immature, pre-neurons. Derivation of daughter dishes from mother cultures via trypsinization resulted in exceedingly pure neuronal cultures devoid of glial cell formation. These cultures contained cells at various stages of maturity, as evidenced by the lesser degree of mature neuronal marker SMI-32R staining as compared with that of β-III-tubulin and neurogenin. This is similarly noted in both the limited presence of double-positive tubulin/GFAP cells [33] along with the high degree of ChAT- and TH-positive neurons, indicating the presence of possibly immature, lineage naïve cells [34].

Neurons isolated from mother cultures displayed normal neuron morphology and functionality, with physiological properties not deviating from exhibited by healthy controls. All neurons examined demonstrated single or multiple membrane potential-dependent spikes with mean durations and amplitudes typical of iPSC-derived neuronal action potentials *in vitro* [35], though kinetics appear slower than typical slice cultures due to room temperature recordings [36]. Furthermore, TTX presence abrogated isolated sodium currents, action potentials, and AP-related fast inward currents resulting from membrane depolarization.

Our findings further demonstrate the neuronal-specificity of our procedure and allow for further comparative pathophysiological studies. Adding to the current body of iPSC-derived neuronal models for neurodegenerative diseases such as AD [13,14,17,23], Huntington’s disease [22], and PD [11,16]), MS patient-derived cells did not differ electrophysiologically or morphologically from that of controls. This may be due to the multifactorial nature of MS, requiring various test assays to reveal disease phenotypes for characterization [19]. Nonetheless, the herein established “disease in a dish” MS model can serve as a suitable platform for disease-specific *in vitro* investigations. These include not only inquiries into neuronal processes and drug repositioning/small molecule screenings [14,17–20], but also studies exploring the functional mechanisms of disease-associated SNPs [37]. As such, the established non-integrative procedure provides a suitable foundation for further exploration into the human- and neuron-specific processes, genomics, and epigenomics of MS.

## Supporting Information

S1 Fig Overview images of HC, MSiPN characterization stainings. Representative pictures of β-III-Tubulin, neurogenin, SMI-32R, ChAT, and TH stainings from Fig 4; scale bar 100 μm.
(TIF)

S2 Fig Representative negative control stainings. (A) Neuronal cultures do not exhibit oligodendrocyte lineage markers a, O4 and b, MBP. (B) Cultures show limited presence of astrocytes, with MSiPNs showing 3.63 (1.22) and HCiPN 0.13 (0.13) percent (SEM) of GFAP-positive, tubulin-negative cells. Error bars depict SEM. (C) a, Negative controls of biotin intermediary with secondary antibodies and b, secondary antibodies alone show limited non-specific staining.
(TIF)

## References

[1] Minagar A, Toledo EG, Alexander JS, Kelley RE. Pathogenesis of Brain and Spinal Cord Atrophy in Multiple Sclerosis. J Neuroimaging. Blackwell Publishing Ltd; 2004;14: 5S–10S. 10.1111/j.1552-6569.2004.tb00273.x 15228754

[2] Friese MA, Schattling B, Fugger L. Mechanisms of neurodegeneration and axonal dysfunction in multiple sclerosis. Nat Rev Neurol. Nature Publishing Group, a division of Macmillan Publishers Limited. All Rights Reserved.; 2014;10: 225–238. http://www.nature.com/nrneurol/journal/v10/n4/abs/nrneurol.2014.37.html#supplementary-information 10.1038/nrneurol.2014.37 24638138

[3] Haghikia A, Hohlfeld R, Gold R, Fugger L. Therapies for multiple sclerosis: translational achievements and outstanding needs. Trends Mol Med. 2013;19: 309–319. 10.1016/j.molmed.2013.03.004 23582699

[4] Takahashi K, Yamanaka S. Induction of pluripotent stem cells from mouse embryonic and adult fibroblast cultures by defined factors. Cell. 2006;126: 663–76. 10.1016/j.cell.2006.07.024 16904174

[5] Aasen T, Raya A, Barrero MJ, Garreta E, Consiglio A, Gonzalez F, et al Efficient and rapid generation of induced pluripotent stem cells from human keratinocytes. Nat Biotechnol. Nature Publishing Group; 2008;26: 1276–84. 10.1038/nbt.1503 18931654

[6] Loh Y-H, Agarwal S, Park I-H, Urbach A, Huo H, Heffner GC, et al Generation of induced pluripotent stem cells from human blood. Blood. American Society of Hematology; 2009;113: 5476–9. 10.1182/blood-2009-02-204800 PMC2689048 19299331

[7] Sun N, Panetta NJ, Gupta DM, Wilson KD, Lee A, Jia F, et al Feeder-free derivation of induced pluripotent stem cells from adult human adipose stem cells. Proc Natl Acad Sci U S A. 2009;106: 15720–5. 10.1073/pnas.0908450106 19805220 PMC2739869

[8] Zhou T, Benda C, Dunzinger S, Huang Y, Ho JC, Yang J, et al Generation of human induced pluripotent stem cells from urine samples. Nat Protoc. 2012;7: 2080–2089. 10.1038/nprot.2012.115 23138349

[9] Yu J, Hu K, Smuga-Otto K, Tian S, Stewart R, Slukvin II, et al Human induced pluripotent stem cells free of vector and transgene sequences. Science. American Association for the Advancement of Science; 2009;324: 797–801. 10.1126/science.1172482 19325077 PMC2758053

[10] Burkhardt MF, Martinez FJ, Wright S, Ramos C, Volfson D, Mason M, et al A cellular model for sporadic ALS using patient-derived induced pluripotent stem cells. Mol Cell Neurosci. 2013;56: 355–364. 10.1016/j.mcn.2013.07.007 23891805 PMC4772428

[11] Byers B, Lee H, Reijo Pera R. Modeling Parkinson’s Disease Using Induced Pluripotent Stem Cells. Curr Neurol Neurosci Rep. Current Science Inc.; 2012;12: 237–242. 10.1007/s11910-012-0270-y 22538490 PMC3342500

[12] Dimos JT, Rodolfa KT, Niakan KK, Weisenthal LM, Mitsumoto H, Chung W, et al Induced pluripotent stem cells generated from patients with ALS can be differentiated into motor neurons. Science (80-). 2008;321: 1218–1221. 10.1126/science.1158799 18669821

[13] Israel MA, Yuan SH, Bardy C, Reyna SM, Mu Y, Herrera C, et al Probing sporadic and familial Alzheimer’s disease using induced pluripotent stem cells. Nature. 2012;482: 216–220. 10.1038/nature10821 22278060 PMC3338985

[14] Kondo T, Asai M, Tsukita K, Kutoku Y, Ohsawa Y, Sunada Y, et al Modeling Alzheimer’s disease with iPSCs reveals stress phenotypes associated with intracellular Aβ and differential drug responsiveness. Cell Stem Cell. 2013;12: 487–496. 10.1016/j.stem.2013.01.009 23434393

[15] Park I-H, Arora N, Huo H, Maherali N, Ahfeldt T, Shimamura A, et al Disease-specific induced pluripotent stem cells. Cell. 2008;134: 877–886. 10.1016/j.cell.2008.07.041 18691744 PMC2633781

[16] Sánchez-Danés A, Richaud-Patin Y, Carballo-Carbajal I, Jiménez-Delgado S, Caig C, Mora S, et al Disease‐specific phenotypes in dopamine neurons from human iPS‐based models of genetic and sporadic Parkinson’s disease. EMBO Mol Med. 2012;4: 380–395. 10.1002/emmm.201200215 22407749 PMC3403296

[17] Yagi T, Ito D, Okada Y, Akamatsu W, Nihei Y, Yoshizaki T, et al Modeling familial Alzheimer’s disease with induced pluripotent stem cells. Hum Mol Genet. 2011;20: 4530–4539. 10.1093/hmg/ddr394 21900357

[18] Cao L, Tan L, Jiang T, Zhu X-C, Yu J-T. Induced Pluripotent Stem Cells for Disease Modeling and Drug Discovery in Neurodegenerative Diseases. Mol Neurobiol. 2014; 1–12. 10.1007/s12035-014-8867-6 25146848

[19] Marchetto MCN, Winner B, Gage FH. Pluripotent stem cells in neurodegenerative and neurodevelopmental diseases. Hum Mol Genet. 2010; ddq159. 10.1093/hmg/ddq159 PMC2875052 20418487

[20] Richard J-P, Maragakis NJ. Induced pluripotent stem cells from ALS patients for disease modeling. Brain Res. 2014; 10.1016/j.brainres.2014.09.017 PMC4362997 25223906

[21] Winner B, Marchetto MC, Winkler J, Gage FH. Human-induced pluripotent stem cells pave the road for a better understanding of motor neuron disease. Hum Mol Genet. 2014; ddu205. 10.1093/hmg/ddu205 24821704

[22] The HD iPSC Consortium. Induced Pluripotent Stem Cells from Patients with Huntington’s Disease Show CAG-Repeat-Expansion-Associated Phenotypes. Cell Stem Cell. 2012;11: 264–278. 10.1016/j.stem.2012.04.027 22748968 PMC3804072

[23] Zhang N, An MC, Montoro D, Ellerby LM. Characterization of human Huntington’s disease cell model from induced pluripotent stem cells. PLoS Curr. 2010;2. 10.1371/currents.RRN1193 PMC2966296 21037797

[24] Qiang L, Fujita R, Abeliovich A. Remodeling neurodegeneration: somatic cell reprogramming-based models of adult neurological disorders. Neuron. 2013;78: 957–969. 10.1016/j.neuron.2013.06.002 23791192

[25] Ingelfinger JR. Nephrogenic Adenomas as Renal Tubular Outposts. N Engl J Med. Massachusetts Medical Society; 2002;347: 684–686. 10.1056/NEJMe020084 12200558

[26] Okita K, Matsumura Y, Sato Y, Okada A, Morizane A, Okamoto S, et al A more efficient method to generate integration-free human iPS cells. Nat Methods. 2011;8: 409–412. 10.1038/nmeth.1591 21460823

[27] Generation of Human Induced Pluripotent Stem Cells (hiPSCs) from Fibroblasts using Episomal Vectors. In: Life Technologies Protocols [Internet]. 2012 [cited 24 Feb 2015]. Available: http://www.lifetechnologies.com/de/de/home/references/protocols/cell-culture/stem-cell-protocols/ipsc-protocols/generation-human-induced-pluripotent-stem-cells-fibroblasts.html

[28] Liu K, Wang F, Ye X, Wang L, Yang J, Zhang J, et al KSR-based medium improves the generation of high-quality mouse iPS cells. PLoS One. 2014;9: e105309 10.1371/journal.pone.0105309 25171101 PMC4149410

[29] Koch P, Opitz T, Steinbeck JA, Ladewig J, Brüstle O. A rosette-type, self-renewing human ES cell-derived neural stem cell with potential for in vitro instruction and synaptic integration. Proc Natl Acad Sci. 2009;106: 3225–3230. 10.1073/pnas.0808387106 19218428 PMC2651316

[30] Martín ED, Buño W. Caffeine-mediated presynaptic long-term potentiation in hippocampal CA1 pyramidal neurons. J Neurophysiol. 2003;89: 3029–38. 10.1152/jn.00601.2002 12783948

[31] Gründken C, Hanske J, Wengel S, Reuter W, Abdulazim A, Shestopalov VI, et al Unified patch clamp protocol for the characterization of Pannexin 1 channels in isolated cells and acute brain slices. J Neurosci Methods. 2011;199: 15–25. 10.1016/j.jneumeth.2011.04.016 21549752

[32] Kim D-WD-S, Lee JS, Leem JW, Huh YJ, Kim JY, Kim H-S, et al Robust enhancement of neural differentiation from human ES and iPS cells regardless of their innate difference in differentiation propensity. Stem Cell Rev Reports. 2010;6: 270–281. 10.1007/s12015-010-9138-1 20376579

[33] Liu Y, Namba T, Liu J, Suzuki R, Shioda S, Seki T. Glial fibrillary acidic protein-expressing neural progenitors give rise to immature neurons via early intermediate progenitors expressing both glial fibrillary acidic protein and neuronal markers in the adult hippocampus. Neuroscience. 2010;166: 241–51. 10.1016/j.neuroscience.2009.12.026 20026190

[34] Ernsberger U, Patzke H, Rohrer H. The developmental expression of choline acetyltransferase (ChAT) and the neuropeptide VIP in chick sympathetic neurons: evidence for different regulatory events in cholinergic differentiation. Mech Dev. 1997;68: 115–126. 10.1016/S0925-4773(97)00135-4 9431809

[35] Karumbayaram S, Novitch BG, Patterson M, Umbach JA, Richter L, Lindgren A, et al Directed differentiation of human-induced pluripotent stem cells generates active motor neurons. Stem Cells. 2009;27: 806–11. 10.1002/stem.31 19350680 PMC2895909

[36] Fitzakerley JL, Schaefer KL, Kitko RA, Manis PB. Properties of cochlear nucleus neurons in primary culture. Hear Res. 1997;114: 148–168. 10.1016/S0378-5955(97)00158-5 9447929

[37] Gregory AP, Dendrou CA, Attfield KE, Haghikia A, Xifara DK, Butter F, et al TNF receptor 1 genetic risk mirrors outcome of anti-TNF therapy in multiple sclerosis. Nature. Nature Publishing Group, a division of Macmillan Publishers Limited. All Rights Reserved.; 2012;488: 508–11. 10.1038/nature11307 22801493 PMC4268493

