# Supplementary figures and images for: Multiple Sclerosis Patient-Specific Primary Neurons Differentiated from Urinary Renal Epithelial Cells via Induced Pluripotent Stem Cells
Source: PLoS One. 2016 May 9;11(5):e0155274. doi: 10.1371/journal.pone.0155274 (PMC4861271; doi:10.1371/journal.pone.0155274)

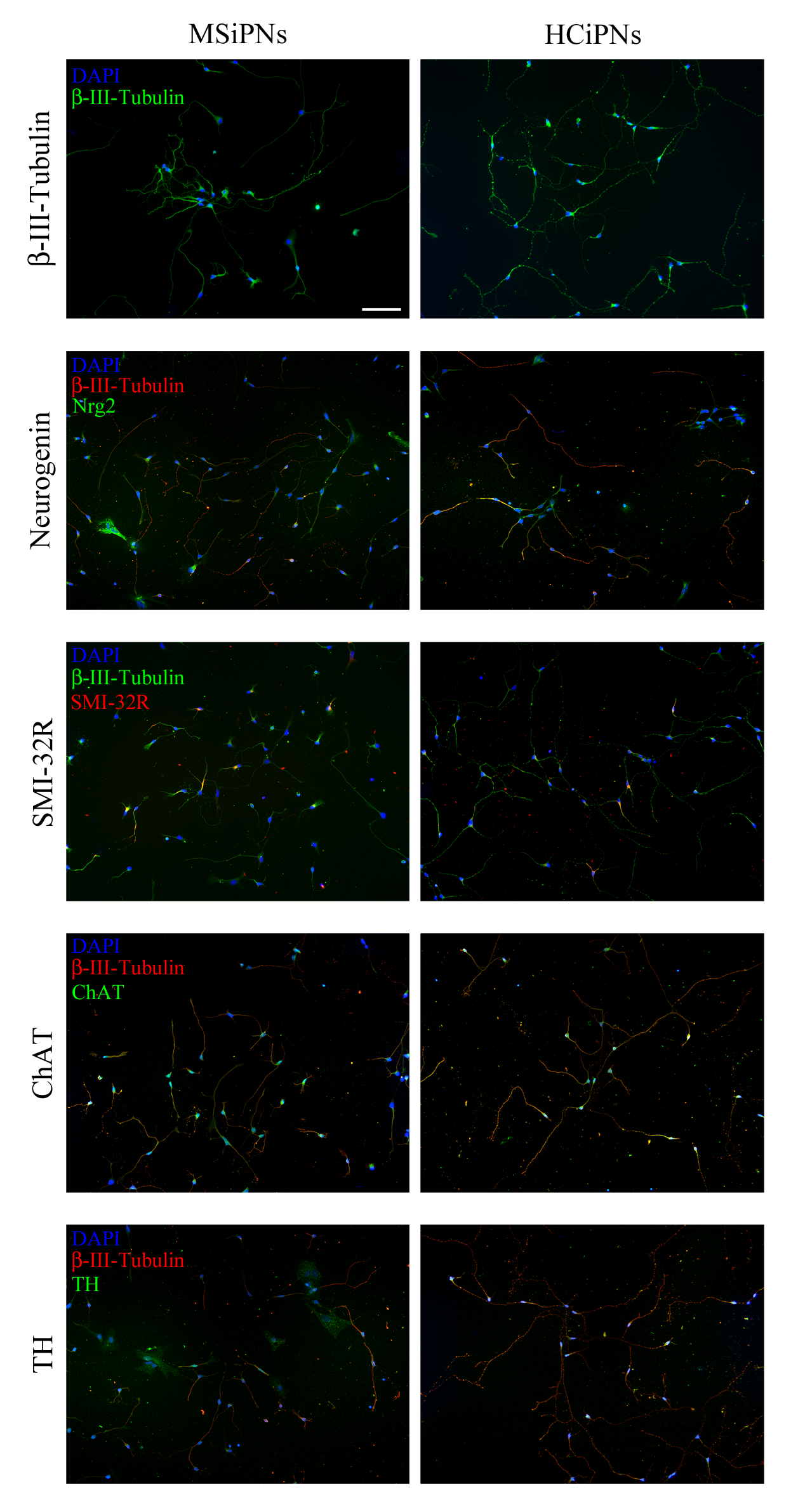

Supplement: S1 Fig — Representative pictures of β-III-Tubulin, neurogenin, SMI-32R, ChAT, and TH stainings from Fig 4; scale bar 100 μm. (TIF) [file pone.0155274.s001.tif]

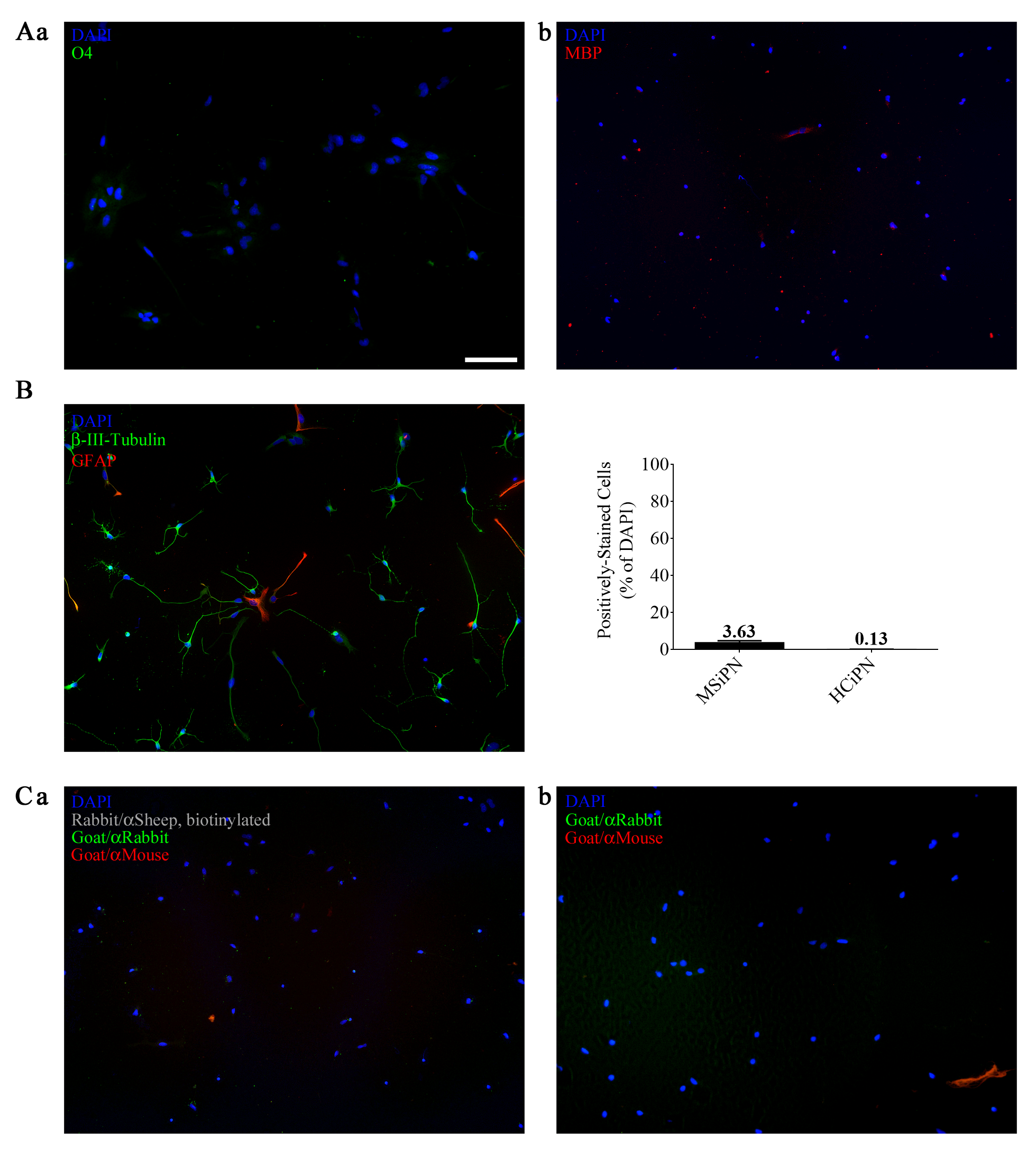

Supplement: S2 Fig — (A) Neuronal cultures do not exhibit oligodendrocyte lineage markers a, O4 and b, MBP. (B) Cultures show limited presence of astrocytes, with MSiPNs showing 3.63 (1.22) and HCiPN 0.13 (0.13) percent (SEM) of GFAP-positive, tubulin-negative cells. Error bars depict SEM. (C) a, Negative controls of biotin intermediary with secondary antibodies and b, secondary antibodies alone show limited non-specific staining. (TIF) [file pone.0155274.s002.tif]
